# Host–parasite coevolution: Partitioning the effects of natural selection and environmental change using coupled Price equations

**DOI:** 10.1002/ece3.9136

**Published:** 2022-07-31

**Authors:** Curtis M. Lively, Michael J. Wade

**Affiliations:** ^1^ Department of Biology Indiana University Bloomington Indiana USA

**Keywords:** eco‐evolutionary feedbacks, fundamental theorem of natural selection, host–parasite coevolution, Price equation

## Abstract

George Price showed how the effects of natural selection and environmental change could be mathematically partitioned. This partitioning may be especially useful for understanding host–parasite coevolution, where each species represents the environment for the other species. Here, we use coupled Price equations to study this kind of antagonistic coevolution. We made the common assumption that parasites must genetically match their host's genotype to avoid detection by the host's self/nonself recognition system, but we allowed for the possibility that non‐matching parasites have some fitness. Our results show how natural selection on one species results in environmental change for the other species. Numerical iterations of the model show that these environmental changes can periodically exceed the changes in mean fitness due to natural selection, as suggested by R.A. Fisher. Taken together, the results give an algebraic dissection of the eco‐evolutionary feedbacks created during host–parasite coevolution.

## Graphical abstract

We used the Price equation to study antagonistic coevolution. The results directly show how natural selection on the parasite reduces host fitness due environmental change, and vice versa.

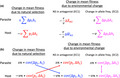

## 1 INTRODUCTION

Fisher's formulation of the fundamental theorem of natural selection had two components (Frank & Slatkin, 1992; Price, 1972). The first component concerned the change in mean fitness due to natural selection, which Fisher showed to be equal to the additive genetic variance for fitness divided by mean fitness (Fisher, 1958). This was Fisher's primary focus (Price, 1972). The second component was the “environment,” which included gene interactions and gene frequencies (Frank & Slatkin, 1992). But, as made clearer by Price (1972), Fisher also argued that natural selection would result in environmental feedbacks that would negatively affect fitness, through the second component of the total change in mean fitness. This second component (environmental deterioration) could erase the change in fitness due to natural selection (Fisher, 1958 p. 45), thereby keeping mean fitness near unity:

Alternatively, we may infer that the organic world in general must tend to acquire just that level of adaptation at which the deterioration of the environment is in some species greater, though in some less, than the rate of improvement by Natural Selection, so as to maintain the general level of adaptation as nearly constant.

In addition, Fisher specifically pointed to the potential role played by biological antagonists in environmental deterioration (Fisher, 1958 p. 41–2):

Probably more important than changes in climate will be the evolutionary changes in progress in associated organisms. As each organism increases in fitness, so will its enemies and competitors increase in fitness. Against the action of Natural Selection in constantly increasing the fitness of every organism, at a rate equal to the genetic variance in fitness which that population maintains, is to be set off the very considerable item of the deterioration of its inorganic and organic environment.

On p. 51, he added:

…changes in the organic environment, including the improvement of enemies and competitors … may be in effect either greater or less than the improvement due to Natural Selection.

Fisher did not, however, quantify the environmental deterioration (Queller, 2017), which we do here for a model of host–parasite coevolution.

Fisher's model was not especially intuitive. Fortunately, George Price later clarified the fundamental theorem. In particular, Price showed how the opposing effects of natural selection and environmental change could be mathematically partitioned (Price, 1972). In summarizing, Price concludes that Fisher's theorem was correct, but also incomprehensible:

Meanwhile I trust that the present paper corrects any diminution in Fisher's mathematical reputation resulting from the common belief that he was seriously mistaken about his theorem. Doubtless this paper also adds considerably to his reputation for incomprehensibility.

Frank and Slatkin (1992) also argued that Fisher's model was fundamentally correct, and they further showed (using a discrete time model) how the effects of natural selection and environmental change could be dissected. They then applied the method to evaluate the effects of natural selection and environmental change on clutch size evolution in birds. In general, the Price/Frank–Slatkin model seems like a very useful way to study eco‐evolutionary feedbacks, which should be common in life‐history evolution (Lively, 2012) and host–parasite interactions. For example, Gandon and Day (2009) used the method to examine different kinds of feedback in host–parasite interactions, including epidemiological feedbacks (due to parasite‐mediated changes in host population density) and genetical feedbacks due to host–parasite coevolution. Our model differs in that we show a more direct connection between the elements of the Price equation for each species, that is, how adaptation by natural selection in one species causes environmental deterioration of the other.

In what follows, we apply the Price equation to a matching‐alleles population genetic model of host–parasite interaction. We partitioned the total change in mean fitness for each species into the effects of natural selection and the effects of environmental degradation caused by genetic changes in the antagonist. Our goal was to determine how host–parasite interactions create feedbacks between natural selection and environmental change. The main finding of our approach is that the second component of the Price equation for the host depends strongly on the first component of the Price equation for the parasite and vice versa.

## 2 MODEL

### 2.1 Partitioning the change in mean fitness

Assuming that fitness is the trait of interest, the Price equation for the total change in fitness is (Frank & Slatkin, 1992; Price, 1972):

(1) $$∆\bar{W}=\frac{\mathrm{var}\left(W\right)}{\bar{W}}+\frac{E\left[W∆W\right]}{\bar{W}}$$

The first term on the right‐hand side (RHS) gives the change in mean fitness due to natural selection ($∆{\bar{W}}_{\mathrm{NS}}$), and the second term on the RHS gives the change in mean fitness due to change in the environment ($∆{\bar{W}}_{\mathrm{EC}}$). The environment is broadly defined to include the internal genetical background as well as the external biotical and abiotic environments. Here, we focus on changes in the biotic environment.

We used Frank and Slatkin's (1992) method to partition the total change in parasite mean fitness ($∆\bar{W})$ into its two components: (i) the change due to natural selection ($∆{\bar{W}}_{\mathrm{NS}})$, and (ii) the change due to environmental change ($∆{\bar{W}}_{\mathrm{EC}})$. The change in parasite mean fitness due to natural selection is

(2) $$∆{\bar{W}}_{\mathrm{NS}}={\bar{W}}^{\prime }|E-\bar{W}|E$$

where ${\bar{W}}^{\prime }|E$ gives mean parasite fitness at time step *t* + 1 given the environment at time *t*; $\bar{W}|E$ is simply $\bar{W}$ at time *t*. Here, *E* is the parasite's environment at time *t*, which is represented by the population genetic state of the host at time *t*. In a coevolutionary interaction, we would expect for the distribution of host genotypes to change over time. The change in mean parasite fitness due to change in the host environment is

(3) $$∆{\bar{W}}_{\mathrm{EC}}={\bar{W}}^{\prime }|E^{\prime }-{\bar{W}}^{\prime }|E$$

where ${\bar{W}}^{\prime }|E^{\prime }$ gives the expected parasite fitness at time *t* + 1 given the frequency of the different host genotypes at time *t* + 1. The total change in parasite mean fitness $∆\bar{W}$ is simply the sum of $∆{\bar{W}}_{\mathrm{NS}}$ and $∆{\bar{W}}_{\mathrm{EC}}$ to give

(4) $$∆\bar{W}={\bar{W}}^{\prime }|E^{\prime }-\bar{W}|E$$

The Price Equation (1) can be derived from Equation (4) (Appendix S1).

### 2.2 Natural selection

#### 2.2.1 Parasites: $∆{\bar{W}}_{\mathrm{NS}}$

For simplicity, we assumed a haploid parasite interacting with a haploid host, where infection was determined by a single locus in both species. We assumed a “matching alleles model” of infection in which each parasite genotype must match its host genotype to evade the host's immune response (Agrawal & Lively, 2002; Otto & Michalakis, 1998). Otherwise, the parasite is detected and attacked by the host's self/nonself recognition system (Burnet, 1971; Grosberg & Hart, 2000). We also assumed that each host contacts a single parasite propagule at random. This means that the probability of a match for the *i* ^th^ parasite genotype is equal to the frequency of the matching allele in the host population (*h*_*i*_). Let *p*_*i*_ be the frequency of the *i* ^*th*^ parasite genotype. Let the fitness of matching parasites be equal to one; and let the fitness of non‐matching parasites be 1 – *s*. As such, the fitness of the *i* ^*th*^ parasite genotype is $W_{i}=h_{i}+\left(1-h_{i}\right)\left(1-s\right)$. For *s* < 1, the parasite is detected but only partially eliminated. Let *n* be the number of alleles, which is the same for host and parasite under the matching alleles model. Under these assumptions, we find in general that

(5) $$\bar{W}|E=\sum_{i=1}^{n}p_{i}W_{i}$$

(6) $$\begin{array}{c} {\bar{W}}^{\prime }|E=\sum_{i=1}^{n}p_{i}^{\prime }W_{i}=\sum_{i=1}^{n}\left(p_{i}+∆p_{i}\right)W_{i}=\sum_{i=1}^{n}p_{i}W_{i}+\sum_{i=1}^{n}∆p_{i}W_{i} \end{array}$$

(7) $$\begin{array}{c} {\bar{W}}^{\prime }|E^{\prime }=\sum_{i=1}^{n}p_{i}^{\prime }W_{i}^{\prime }=\sum_{i=1}^{n}\left(p_{i}+∆p_{i}\right)\left(W_{i}+∆W_{i}\right) \end{array}$$

The change in parasite mean fitness due to natural selection reduces to

(8) $$∆{\bar{W}}_{\mathrm{NS}}={\bar{W}}^{\prime }\;\left|\;E-\bar{W}\;\right|\;E=\sum_{i=1}^{n}∆p_{i}W_{i}$$

where $\sum ∆p_{i}W_{i}=\text{var}\left(W\right)/\bar{W}$ (see Appendix S1), which is consistent with the fundamental theorem of natural selection (Fisher, 1918).

Substituting for *W*_*i*_ in Equation (8), we get (since $\sum ∆p_{i}=0),$

(9) $$∆{\bar{W}}_{\mathrm{NS}}=\sum_{i=1}^{n}∆p_{i}\left(1-s\left(1-h_{i}\right)\right)=s\sum_{i=1}^{n}∆p_{i}h_{i}$$

The RHS of Equation (9) can also be written as a covariance (as shown in Appendix S1) to give,

(10) $$∆{\bar{W}}_{\mathrm{NS}}=\mathrm{sn}*\mathrm{cov}\left(∆p_{i},h_{i}\right)$$

Hence, the change in parasite mean fitness due to natural selection depends on the covariance between $∆p_{i}$ and $h_{i}$, which is expected to be positive.

#### 2.2.2 Hosts: $∆{\bar{X}}_{\mathrm{NS}}$

Let *X*_*i*_ be the fitness of the *i* ^th^ host genotype, and let $\bar{X}$ be mean host fitness. Let *E* now stands for the host's environment. As previously *n* is the number of alleles. Following the methods above for the parasite population, we get:

(11) $$\bar{X}|E=\sum_{i=1}^{n}h_{i}X_{i}$$

(12) $$\begin{array}{c} {\bar{X}}^{\prime }|E=\sum_{i=1}^{n}h_{i}^{\prime }X_{i}=\sum_{i=1}^{n}\left(h_{i}+∆h_{i}\right)X_{i}=\sum_{i=1}^{n}h_{i}X_{i}+\sum_{i=1}^{n}∆h_{i}X_{i} \end{array}$$

(13) $$\begin{array}{c} {\bar{X}}^{\prime }|E^{\prime }=\sum_{i=1}^{n}h_{i}^{\prime }X_{i}^{\prime }=\sum_{i=1}^{n}\left(h_{i}+∆h_{i}\right)\left(X_{i}+∆X_{i}\right) \end{array}$$

The change in host mean fitness due to natural selection reduces to

(14) $$∆{\bar{X}}_{\mathrm{NS}}={\bar{X}}^{\prime }|E-\bar{X}|E=\sum_{i=1}^{n}∆h_{i}X_{i}$$

where $\sum ∆h_{i}X_{i}=\text{var}\left(X\right)/\bar{X}$ (see Appendix S1). Assuming a matching alleles model of infection genetics, hosts that are not matched have a relative fitness of 1, while hosts that encounter a matching parasite genotype have a relative fitness of (1−v). The variable v gives the reduction in host fitness due to infection (i.e., virulence). The fitness of host genotype *i* (*X*_*i*_) is then $X_{i}=\left(1-p_{i}\right)+p_{i}\left(1-v\right)$. Thus, the change in host fitness due to natural selection is

(15) $$∆{\bar{X}}_{\mathrm{NS}}=\sum_{i=1}^{n}∆h_{i}\left(1-p_{i}v\right)=\sum_{i=1}^{n}∆h_{i}-v\sum_{i=1}^{n}p_{i}∆h_{i}=-v\sum_{i=1}^{n}p_{i}∆h_{i}$$

(Note that the sum of host–genotype frequency changes is zero [i.e., ∑∆h=0]). The change in host fitness due to natural selection can also be written as a covariance (Appendix S1),

(16) $$∆{\bar{X}}_{\mathrm{NS}}=-\mathrm{vn}*\mathrm{cov}\left(p_{i},∆h_{i}\right)$$

This later result shows that the change in host mean fitness depends on the covariance between $∆h_{i}$ and $p_{i}$, which is expected to be negative if infection reduces host fitness. The negative covariance term gives a positive value when multiplied by −v.

### 2.3 Environmental change

#### 2.3.1 Parasites: $∆{\bar{W}}_{\mathrm{EC}}$

The change in parasite mean fitness due to environmental (host) change is:

(17) $$\begin{array}{c} ∆{\bar{W}}_{\mathrm{EC}}={\bar{W}}^{\prime }\;\left|\;E^{\prime }-{\bar{W}}^{\prime }\;\right|\;E=\sum_{i=1}^{n}p_{i}∆W_{i}+\sum_{i=1}^{n}∆p_{i}∆W_{i} \end{array}$$

(18) $$∆{\bar{W}}_{\mathrm{EC}}=s\sum_{i=1}^{n}p_{i}∆h_{i}+s\sum_{i=1}^{n}∆p_{i}∆h_{i}$$

Importantly, the first term on the RHS of Equation (18) $\left(s\sum p_{i}∆h_{i}\right)\;$ is equal to $-\frac{s}{v}∆{\bar{X}}_{\mathrm{NS}}$ (see Equation (15)), hence we get

(19) $$∆{\bar{W}}_{\mathrm{EC}}=-\frac{s}{v}∆{\bar{X}}_{\mathrm{NS}}+s\sum_{i=1}^{n}∆p_{i}∆h_{i}$$

Note that the first term on the right‐hand side $\left(-\frac{s}{v}∆{\bar{X}}_{\mathrm{NS}}\right)$ shows how parasite‐mediated natural selection on the host feeds back to reduce parasite mean fitness. The magnitude of the effect depends strongly on the strength of selection against mismatched parasites (*s*), where higher values of *s* result in stronger negative feedbacks. Substituting the results of Equation (16) for $∆{\bar{X}}_{\mathrm{NS}}$ into Equation (19), we can rewrite the first term on the RHS as a covariance, giving:

(20) $$∆{\bar{W}}_{\mathrm{EC}}=\mathrm{sn}*\mathrm{cov}\left(p_{i},∆h_{i}\right)+s\sum_{i=1}^{n}∆p_{i}∆h_{i}$$

The total change in mean fitness due to environmental change term also contains a second term, which can be rewritten as (see Appendix S1):

(21) $$s\sum_{i=1}^{n}∆p_{i}∆h_{i}=\mathrm{sn}*\mathrm{cov}\left(∆p_{i},∆h_{i}\right)$$

Hence, the change in parasite fitness due to change in the environment (hosts) is given by

(22) $$∆{\bar{W}}_{\mathrm{EC}}=\mathrm{sn}\left(\mathrm{cov}\left(p_{i},∆h_{i}\right)+\mathrm{cov}\left(∆p_{i},∆h_{i}\right)\right)$$

**FIGURE 1:**
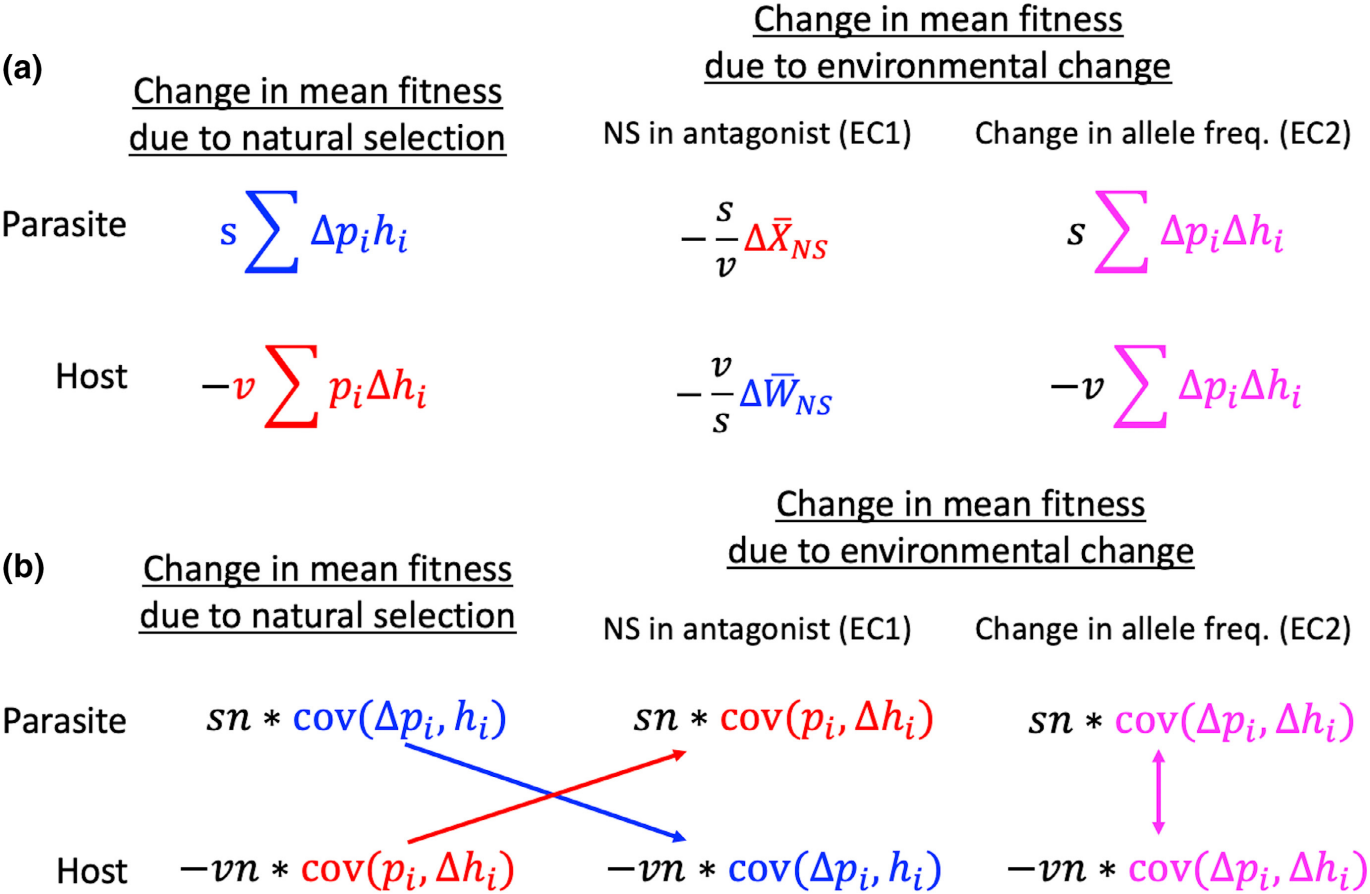
Partitioning the effects of change due to natural selection and to environmental change on the total change in mean fitness for both parasites and hosts: (a) (top), summation approach; (b) (bottom), covariance approach. The environmental change part contains two terms: (1) change due to natural selection in the antagonist (called EC1) and (2) change due to the covariance between changes in the frequencies of the matching alleles (called EC2). Host changes are weighted by v, which is the reduction in fitness caused by infection (i.e., virulence), and parasite changes are weighted by *s*, which is the selection against mismatched parasites. *n *is the number of alleles in both hosts and parasites; *p*_*i*_ is the frequency of the *i* ^th^ allele in the parasite population; and *h*_*i*_ is the frequency of the *i* ^th^ allele in the host population (the subscripts were dropped to simplify the figure). Note how natural selection on the parasite causes environmental change in the host, and vice versa. Equivalent terms within a and within b are indicated by the same colors.

A summary of the results is given in Figure 1.

#### 2.3.2 Hosts: $∆{\bar{X}}_{\mathrm{EC}}$

Similarly, the change in host mean fitness due to environment change is:

$$\begin{array}{c} ∆{\bar{X}}_{\mathrm{EC}}={\bar{X}}^{\prime }\;\left|\;E^{\prime }-{\bar{X}}^{\prime }\;\right|\;E=-v\sum_{i=1}^{n}∆p_{i}h_{i}^{\prime }=-v\sum_{i=1}^{n}∆p_{i}\left(h_{i}+∆h_{i}\right) \end{array}$$

(23) $$∆{\bar{X}}_{\mathrm{EC}}=-v\sum_{i=1}^{n}∆p_{i}h_{i}-v\sum_{i=1}^{n}∆p_{i}∆h_{i}$$

The first term on the RHS of (23) equals the parasite's change in mean fitness owing to natural selection multiplied by $-\frac{v}{s}$ (see Equation (9)), hence

(24) $$∆{\bar{X}}_{\mathrm{EC}}=-\frac{v}{s}∆{\bar{W}}_{\mathrm{NS}}-v\sum_{i=1}^{n}∆p_{i}∆h_{i}$$

This result shows how the change in host mean fitness is directly related to host‐mediated natural selection on the parasite. Substituting the results from Equation (10) for parasite $∆{\bar{W}}_{\mathrm{NS}}$ and substituting from Equation (21) for $\sum ∆p_{i}∆h_{i}$, we get:

(25) $$∆{\bar{X}}_{EC}=-\mathrm{vn}\left(\mathrm{cov}\left(∆p_{i},h_{i}\right)+\mathrm{cov}\left(∆p_{i},∆h_{i}\right)\right)$$

Similar to the results above, the total change in mean fitness due to environmental change for the host also contains a second term ($\mathrm{cov}\left(∆p_{i},∆h_{i}\right)$), which depends on the covariance in allele frequency changes between both species. To determine the relative contributions of the different components over time, we conducted numerical iterations.

## 3 NUMERICAL ITERATIONS

**FIGURE 2:**
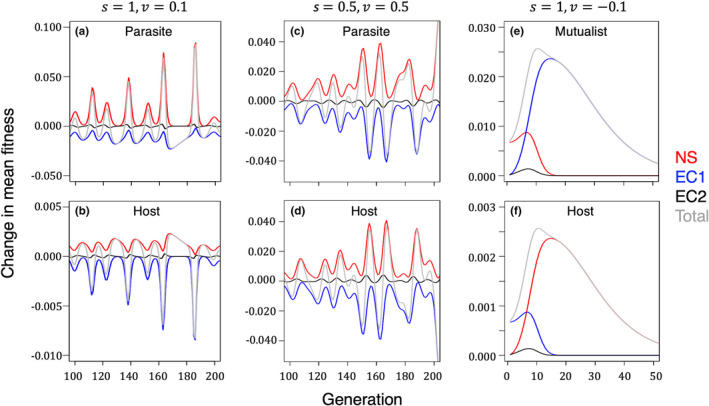
Results of numerical iterations. For panels a and b, we set v = 0.1 and s = 1.0, meaning that mismatched parasites were killed by the host immune system as commonly assumed in matching alleles models. Panel a shows the changes in mean parasite fitness due to natural selection (red line), the change to due natural selection in the host (EC1, blue line), the change due to the covariance in allele frequency changes (EC2, black line) and the total change (gray line). The total change is the sum of NS, EC1 and EC2. Panel b shows the changes in the host population. Note that the negative effect of EC1 on mean fitness periodically exceeds the positive change due to natural selection, resulting in a negative total change in mean fitness. Also note that the effect of EC2 is small relative to EC1. For panels c and d, we set or v=s=0.5, so selection against mismatched parasites was equal to selection against matched host. Note that here the change in mean fitness due to natural selection is equal to the change in mean fitness due to selection on the antagonist. For panels e and f, we set v=−0.1,s=1, making the relationship mutualistic rather that parasitic. Here, the change due to environmental is always positive, and that it can also exceed the direct change due to natural selection in either the parasite (panel e) or the host (panel f). Here, the changes converge on zero as the genetic variance in the host and parasite is eroded. As observed for parasites, the contribution of EC2 was small.

To compare the different components for change in mean fitness over time, we conducted numerical iterations of the equations in Figure 2. The iterations (using R, [“R: A language and environment for statistical computing,” R Core Team, 2019]) assumed four genotypes for both host and parasite, in which both the hosts and parasites made random contact. Fitnesses were calculated based on the probabilities of matching, as outlined above. The program ran for 200 generations, and the changes in mean fitness due to natural selection and environmental change were calculated. We used the results to determine the relative effects of natural selection and environmental change on both the parasite and the host (Figures 2a–d). We also examined these changes when the symbiont had a positive, rather than a negative, effect on the host, thus making the symbiont a mutualist (Figures 2e,f).

With respect to host–parasite coevolution, the results show how the changes due to natural selection and environmental change fluctuate over time. The results also show that environmental change in the parasite due to natural selection on the host can outweigh the direct effects of natural selection on the parasite during parts of the co‐evolutionary cycle (Figures 2a,c). The converse was also true for the host (Figures 2b,d). Finally, the results suggest that the second environmental change term (resulting from $\mathrm{cov}\left(∆p_{i},∆h_{i}\right)$) is small. Thus, by far, the largest contribution to environmental change comes from natural selection on the antagonist.

With respect to a mutualistic interaction, the results again showed that environmental change can exceed the effect of natural selection on mean fitness for both species (Figures 2e,f). But here the effect of environmental change was positive rather than negative, leading to fixation of alleles, rather than to the oscillatory dynamics seen for host–parasite coevolution. Finally, the second term for environmental change played a relatively small role in dictating the total change in mean fitness for the mutualist pair, as was also observed for host–parasite coevolution.

## 4 DISCUSSION

The Price equation is a powerful way to study eco‐evolutionary feedbacks. For example, Frank and Slatkin (1992) provided a discrete time formulation of the Price equation to partition the effects of natural selection and environmental change, which they used to study clutch size evolution in birds. Lively (2012) used the method to study Fisher's idea that environmental deterioration could counter the gains in mean fitness due to fecundity selection. The results showed that the change in mean fitness due to environmental change (increased density) is a negative mirror image of the positive effects of natural selection on mean fitness, where the two effects almost exactly cancel each other. Gandon and Day (2009) used the method to study host–parasite interactions, including coevolution with specificity for infection. In particular, they showed that the change in mean parasite fitness due to environmental change depends on the change in the frequency of host strains that are susceptible to different parasite strains and vice versa (see section 2.2 of supplementary material for Gandon and Day (2009)).

The present paper also uses the Price equation to study host–parasite (or mutualist) coevolution. Our primary goal was to understand the effect of natural selection in one species on the environmental change in the antagonist. We assumed that parasites must genetically match their hosts to avoid detection by the host immune system, but we allowed for the possibility that non‐matching parasites had some fraction of the fitness expected for matching parasites. The basic model is based on the self/nonself recognition systems in animals (e.g., Burnet, 1971; Grosberg & Hart, 2000), which is the most commonly used assumption in theoretical studies of host–parasite coevolution (e.g., Frank, 1993; Gandon & Day, 2009; Hamilton, 1980, 1993; Howard & Lively, 1994; Nee, 1989; Otto & Michalakis, 1998).

### 4.1 Change due to natural selection

As expected, the results were consistent with the fundamental theorem of natural selection: the change in mean fitness due to natural selection is equal to the additive genetic variance for fitness divided by mean fitness (see also Gandon & Day, 2009). This is true for both the host and the parasite. The results further show that the change in mean fitness due to natural selection on the parasite depends on the covariance between the change in parasite allele frequency and the frequency of the matching host genotype (Equation 10). Similarly, the change in mean fitness due to natural selection on the host depends on the covariance between the change in host allele frequency and the frequency of the matching allele in the parasite population (Equation 16). The results thus show how the variance in relative fitness for both antagonists depends on the covariance between the change in allele frequencies and the frequency of the matching allele in the other species: for example, $∆{\bar{W}}_{\mathrm{NS}}=\frac{\mathrm{var}\left(W\right)}{\bar{W}}=\mathrm{sn}*\mathrm{cov}\left(∆p_{i},h_{i}\right)$.

### 4.2 Change due to environmental deterioration

The change in mean fitness due to environmental deterioration is more complicated, as it is the sum of two parts. The first part is negative for both species, and it depends on the change in mean fitness due to natural selection in the antagonist (EC1 in Figure 2). For example, parasite‐mediated natural selection on the host leads directly to environmental change for the parasite. The magnitude of the effect is strongly influenced by the relative values for virulence (*v*) and selection against mismatched parasites (*s*). For example, increasing *s* relative to *v* increases the effect of environmental change for parasites resulting from natural selection on the host. In contrast, increasing *s* relative to *v* decreases the effect of environmental change for hosts resulting from natural selection on the parasite. Note that for the special case of *v* = *s*, the first environmental change term (EC1) is equal to the change in mean fitness in the antagonist. An illustration of this result is given in Figures 2c,d.

The second part of the environmental change term (EC2) depends on the covariance between changes in allele frequencies (Figure 1). This term is like an interspecific linkage disequilibrium, as it specifies how change at the parasite locus covaries with the change at the host locus. A positive covariance has a positive effect on mean fitness in the parasite, and a negative effect in the host (Figure 1). However, the numerical iterations suggest that the term is small, at least when compared to the change due to natural selection in the antagonist. This finding of a relatively small effect held for both parasites and mutualists (Figure 2). The term may be small as there is no means in the present formulation for transmitting interspecific associations built up by selection within generations.

### 4.3 Numerical iterations: Comparing the components for change over time

To study different components for change in mean fitness over time, we first set *v* = 0.1 and *s* = 1. As such, the relative fitness of a matched host was equal to 1 – *v* = 0.9, and the relative fitness of a mismatched parasite was 1 – *s* = 0. We found that the change in mean fitness due to natural selection on the parasite was periodically smaller than the environmental change in mean fitness in the parasite that was due to natural selection on the antagonist. This was true for both the parasite (Figure 2a) and the host (Figure 2b). A similar result was observed for the case of symmetrical effects of infection on both species (*s* = *v*) (Figures 2c,d), suggesting that the previous result (for *s* > *v*) did not stem from stronger selection on the parasite. Taken together, these results give motivation for keeping the second term of the Price equation when investigating coevolutionary interactions. This finding contrasts with the effects of the intrinsic genetic environment in which the second term of the Price equation can be very small (Queller, 2017).

The present model also allows for the possibility that the “parasite” is instead a mutualist, simply by setting the virulence term, *v*, to a negative value. In this case, the change in mean fitness due to environmental change is positive, thereby creating a positive, rather than negative, feedback between the symbionts (Figure 1). This kind of interaction led to a rapid erosion of genetic variance in both species. And, as in the case for parasites, the change in fitness due to selection in the antagonist could outweigh the direct change due to natural selection on the target species. Finally, as observed for parasites, the second part of the environmental change term had a small effect relative to the change in fitness due to natural selection on the other mutualist (Figure 2e,f).

### 4.4 Conclusions and caveats

Fishers' fundamental theorem of natural selection was originally misunderstood, owing in part to Fisher's presentation. Many theoretical geneticists including Kimura (1958, p. 166), Turner (1967), Crow and Kimura (1956), and Crow and Kimura (1970, p. 214–15) have investigated the relationship between natural selection and mean population fitness. Whereas Turner (1967) elected to ignore the effects of environment, Kimura (1958) and Crow and Kimura (1970) attempted to partition the effects of gene interaction (i.e., dominance and epistasis) away from other, non‐genic environmental effects in order to more cleanly separate genetic from environmental effects that Fisher had lumped together into a single environmental term. For example, Crow and Kimura (1970, p. 210) cite Fisher (1958, p. 41 see above) and then model “the effects of overcrowding and deterioration of the environment.” Following Kimura (1958, p.168), they derive equation 5.6.15 (p. 214), partitioning the change in mean fitness into three terms: the genic variance, the change in genotypic fitness owing to change in the environment, and a third term representing deviations from Hardy–Weinberg and gene interactions. They interpret the second term in a manner similar to Price (1972) as “*In a natural population, the environment is continually deteriorating, primarily because of the improvement of competing species. This term can be thought of as a measure of such deterioration*.” (Crow & Kimura, 1970, p. 210). This mathematical decoupling of the effects of natural selection and environmental change (now called the Price equation) may be especially useful in studies of host–parasite coevolution, where each species represents an essential aspect of the environment for the other species.

Our study shows that the change in mean fitness due to environmental change depends directly on the change in mean fitness due to natural selection in the antagonist, plus an additional small effect due to the covariance in frequency changes between matching genotypes established by selection. As such, the model provides a heuristic framework for understanding the statistical genetics underlying the feedbacks that can occur during host–parasite coevolution. Nonetheless, the present formulation relies on simplifying assumptions regarding the infection matrix (matching alleles), the genetic basis of resistance (single locus, haploid), and population size (large, no genetic drift). Relaxing these simplifying assumptions would likely add additional terms to the solution (e.g., Gandon & Day, 2009), but it seems reasonable to suspect that the conceptual framework would remain intact.

## AUTHOR CONTRIBUTIONS

**Curtis M. Lively:** Conceptualization (equal); formal analysis (equal); software (lead); writing – original draft (lead). **Michael J. Wade:** Conceptualization (equal); formal analysis (equal).

## CONFLICT OF INTEREST

We have no conflicts of interest to declare.

## Supporting information


Appendix S1

## Data Availability

The computer code for the numerical iterations (as an R file, and as a text file), along with a README file (txt) defining the variables, has been uploaded to Zenodo: https://doi.org/10.5281/zenodo.6802223.
