# Supplementary material for: Host–parasite coevolution: Partitioning the effects of natural selection and environmental change using coupled Price equations
Source: Ecol Evol. 2022 Jul 31;12(8):e9136. doi: 10.1002/ece3.9136 (PMC9339760; doi:10.1002/ece3.9136)
Supplement: Supplementary file 1 — Appendix S1 [file ECE3-12-e9136-s001.docx]

**Appendix A**. One reviewer suggested that we show here how the Price equation can be derived from equation (4). Beginning with equation (4), the derivation can be done as follows.

$$\Delta\bar{W}=\bar{W}^{'}|E^{'}-\bar{W}|E$$

$$\Delta\bar{W}=\sum_{i=1}^{n} p_{i}^{'}W_{i}^{'}-\sum_{i=1}^{n} p_{i}W_{i}$$

Dropping the subscripts, we get.

$$\Delta\bar{W}=\sum p^{'}(W+\Delta W)-\sum pW$$

$$\Delta\bar{W}=\sum\frac{pW}{\bar{W}}(W+\Delta W)-\bar{W}$$

$$\Delta\bar{W}=\sum\frac{pW^{2}}{\bar{W}}+\sum\frac{pW\Delta W}{\bar{W}}-\bar{W}$$

$$\Delta\bar{W}=\bar{\frac{W^{2}}{\bar{W}}}+\frac{E[W\Delta W]}{\bar{W}}-\bar{W}$$

$$\Delta\bar{W}=\bar{\frac{W^{2}}{\bar{W}}}-\frac{\bar{W}^{2}}{\bar{W}}+\frac{E[W\Delta W]}{\bar{W}}$$

Note that $\bar{W^{2}}-\bar{W}^{2}=\mathrm{var}(W)$. Hence, we get

$$\Delta\bar{W}=\frac{\mathrm{var}(W)}{\bar{W}}+\frac{E[W\Delta W]}{\bar{W}}$$

The last step gives the Price equation, where the first term on the RHS is the change due to natural selection and the second term on the RHS is the change due to change in the environment. See Price (1972) for the original derivation (see also Frank, 1997; Gardner, 2008; Lynch & Walsh, 1998). For the derivation of similar results prior to Price’s (1972) paper, see Crow and Kimura (1970). Finally, the extension of equation (8) given in the text can be given as

$$\Delta\bar{W}_{NS}=\sum_{i=1}^{n} \Delta p_{i}W_{i}$$

Dropping the subscripts, we get

$$\Delta\bar{W}_{NS}=\sum(p^{'}-p)W$$

$$\Delta\bar{W}_{NS}=\sum(\frac{pW}{\bar{W}}-p)W$$

$$\Delta\bar{W}_{NS}=\sum(\frac{pW^{2}}{\bar{W}}-pW)$$

$$\Delta\bar{W}_{NS}=\frac{\bar{W}^{2}}{\bar{W}}-\bar{W}$$

$$\Delta\bar{W}_{NS}=\frac{\mathrm{var}(W)}{\bar{W}}$$

The same protocol was used to derive equation (14).

**Appendix B**. Derivation of equation (10).

From equation (9), we have $\Delta\bar{W}_{NS}=s\sum_{i=1}^{n} \Delta p_{i}h_{i}$

Dropping the subscripts and multiplying both sides by $\frac{1}{sn}$, we get: $\frac{1}{sn}\Delta\bar{W}_{NS}=\frac{1}{n}\sum\Delta ph$

Subtracting $\bar{\Delta p} \bar{h}$ from both sides, we get: $\frac{1}{sn}\Delta\bar{W}_{NS}-\bar{\Delta p} \bar{h}=\frac{1}{n}\sum\Delta ph-\bar{\Delta p} \bar{h}$

Given that $\frac{1}{n}\sum\Delta ph-\bar{\Delta p} \bar{h}=\mathrm{cov}(\Delta p, h)$, we get $\frac{1}{sn}\Delta\bar{W}_{NS}-\bar{\Delta p} \bar{h}=\mathrm{cov}(\Delta p, h)$

Given that $\bar{\Delta p}=0$, we get $\Delta\bar{W}_{NS}=sn*\mathrm{cov}(\Delta p, h)$, which is equation (10).

The derivations for equations (16) and (21) follow the same protocol.
